# Reweighted Off-Grid Sparse Spectrum Fitting for DOA Estimation in Sensor Array with Unknown Mutual Coupling

**DOI:** 10.3390/s23136196

**Published:** 2023-07-06

**Authors:** Liangliang Li, Xianpeng Wang, Xiang Lan, Gang Xu, Liangtian Wan

**Affiliations:** 1 State Key Laboratory of Marine Resource Utilization in South China Sea, School of Information and Communication Engineering, Hainan University, Haikou 570228, China; liangliangli0717@126.com (L.L.); xlan@hainanu.edu.cn (X.L.); 2 State Key Laboratory of Millimeter Waves, Southeast University, Nanjing 210096, China; gangxu@seu.edu.cn; 3 Key Laboratory for Ubiquitous Network and Service Software of Liaoning Province, School of Software, Dalian University of Technology, Dalian 116620, China; wanliangtian@dlut.edu.cn

**Keywords:** DOA estimation, sensor array, unknown mutual coupling, off-grid error, Sparse Spectrum Fitting, reweighted sparse recovery

## Abstract

In the environment of unknown mutual coupling, many works on direction-of-arrival (DOA) estimation with sensor array are prone to performance degradation or even failure. Moreover, there are few literatures on off-grid direction finding using regularized sparse recovery technology. Therefore, the scenario of off-grid DOA estimation in sensor array with unknown mutual coupling is investigated, and then a reweighted off-grid Sparse Spectrum Fitting (Re-OGSpSF) approach is developed in this article. Inspired by the selection matrix, an undisturbed array output is formed to remove the unknown mutual coupling effect. Subsequently, a refined off-grid SpSF (OGSpSF) recovery model is structured by integrating the off-grid error term obtained from the first-order Taylor approximation of the higher-order term into the underlying on-grid sparse representation model. After that, a novel Re-OGSpSF framework is formulated to recover the sparse vectors, where a weighted matrix is developed by the MUSIC-like spectrum function to enhance the solution’s sparsity. Ultimately, off-grid DOA estimation can be realized with the help of the recovered sparse vectors. Thanks to the off-grid representation and reweighted strategy, the proposed method can effectively and efficiently achieve high-precision continuous DOA estimation, making it favorable for real-time direction finding. The simulation results validate the superiority of the proposed method.

## 1. Introduction

Parameter estimation has drawn widespread concern and become a research hotspot in the field of array signal processing over the past few decades, especially for direction-of-arrival (DOA) estimation [1,2]. To the best of our knowledge, DOA estimation mainly uses sensor array to sample, analyze, and process spatial signals, achieving azimuth and elevation angles estimation for interested targets. It is one of the research foundations and vital components of parameter estimation, which provides precious angle information, and even prepares for subsequent parameter information like position. It is generally encountered in various real-life applications, such as unmanned aerial vehicles (UAV), vehicle localization, navigation, etc [3,4].

Currently, these efforts towards direction finding can be roughly grouped into two categories, i.e., subspace technologies [5,6,7,8] and sparse signal recovery (SSR) attempts [9,10,11,12,13]. The former are represented by the multiple signal classification (MUSIC) algorithm [5] and the estimation of signal parameters via rotational invariance techniques (ESPRIT) algorithm [6], officially opening the era of super-resolution direction finding. Afterwards, subspace estimators [7,8] are successively refined from different aspects, such as accuracy and robustness. These subspace frameworks largely rest with the eigenvalue decomposition (EVD) of the covariance matrix to decouple the subspaces, and then exploit the inherent relationships between the array manifolds and the decoupled subspaces to estimate DOAs. However, they are quite susceptible to the effects of signal-to-noise ratio (SNR), snapshots number, coherent targets, etc. In other words, it is hard for them to obtain the desirable performance under relatively harsh direction finding circumstances, such as unsatisfactory SNR and insufficient snapshots. Motivated by the potential spatial sparsity of the targets, the SSR perspective based on the principle of Compressed Sensing (CS) [14,15] came into being to solve the above problem. Subsequently, a set of sparsity-aware estimators are structured, including convex optimization attempts [9,10,11] and sparse Bayesian learning (SBL) efforts [12,13]. Simultaneously, plenty of research results have shown that this not only improves the estimation accuracy under the conditions of undesirable SNR and snapshots, but also enhances the robustness to coherent sources [16].

In terms of SBL-based approaches, their estimation accuracy relies heavily on the discretization degree of the investigated spatial domain. The higher the discretization degree (i.e., the denser the grid points), the smaller the grid interval, and the heavier the computational burden, while the lower the discretization degree (i.e., the sparser the grid points), the larger the grid interval, and the greater the mismatches between the desired DOAs and the closest candidate directions. Since it is almost impossible to ensure that all sources fall precisely on the predefined candidate directions, it is hard for the targets to avoid off-grid error caused by such mismatch.

Many attempts have been made in view of off-grid DOA estimation [17,18,19,20,21]. A Sparse Spectrum Fitting with Modeling Uncertainty (SpSFMU) scheme [17] is developed by linearly approximating the off-grid gap in the closest candidate grid points. Compared to its on-grid SpSF framework, it allows continuous DOA estimation, enhancing the estimation accuracy or/and relieving the computational load. Inspired by the linear approximation, a novel off-grid sparse Bayesian inference (OGSBI) approach [18] is presented with the assumption that the off-grid gap is uniformly distributed within the grid interval, where the off-grid gap is ultimately calculated by the expectation maximization (EM) strategy. As shown in [19], a robust block-sparse Bayesian learning framework without noise variance estimation is further derived via employing the sample covariance matrix. Despite these approaches [18,19] being robust to off-grid gap, it is computationally expensive for them to achieve satisfactory estimation accuracy, and their performance remains unacceptable under the condition of very coarse grids. For realizing a satisfactory performance under the coarse grid condition without aggravating the computational load, a robust root off-grid sparse Bayesian learning (Root-OGSBL) method [20] is designed, which dynamically upgrades predefined grid points via solving a polynomial to reduce off-grid error. Inspired by [20], a modified off-grid SBL (Re-OGSBL) approach [21] with a forgotten factor scheme is reported to further refine the dynamic update procedure for grid points.

Although these attempts have some attractive properties, they all either explicitly or implicitly require ideal array manifolds. In reality, there are plenty of array manifold perturbations in complex circumstances, such as unknown mutual coupling [22,23] and gain-phase error [24,25]. It should be noted that for a selected array, the more antennas, the smaller their spacing, and the more likely to cause space electromagnetic fields interaction between sensors. In this way, closely-spaced antennas are greatly vulnerable to unknown mutual coupling effect, destroying the desirable array manifold structure and making these approaches damaged or invalid in complex electromagnetic environments.

Therefore, many efforts have been devoted to DOA estimation in unknown mutual coupling [26,27,28,29,30,31,32,33]. Motivated by the spirit of array compensation, a group of auxiliary antennas are additionally placed on the two boundaries of the initial array to avoid the unknown mutual coupling influence, facilitating the direct utilization of the MUSIC principle [26]. Different from [26], a specific selection matrix [27] is structured to achieve array compensation through choosing sensors at the ends of the original array to be auxiliary ones. This attempt provides acceptable direction finding performance with low computational complexity, although at the cost of array aperture. Subsequently, the parameterized decoupling work [28] is reported to decouple the DOA information from the unknown mutual coupling. Despite preserving the entire array aperture well, it leads to a high computational burden. Except for the attempts achieved by subspace techniques in [26,27,28], a series of decoupling investigations on unknown mutual coupling interference have been conducted from the perspective of SSR [29,30,31,32,33]. As discussed in [29], an enhanced $l_{1}$-SVD (singular value decomposition) approach is introduced via using a selection matrix. Afterwards, a block sparse recovery (BSR) estimator [30] is developed in the data domain by parameterizing the coupled array manifold. Following the idea of parameterized decoupling, a robust BSR framework of array covariance vectors is reported as well [31]. However, it is still subject to the $l_{1}$-norm approximation, causing limited recovery performance. Afterwards, weighting techniques have received keen attention for their wide applications in parameter estimation [32,33], data fusion [34,35], error estimation [36,37], etc. Among them, weighting research on DOA estimation, i.e., the reweighted BSR approaches [32,33], are performed to enforce the solution’s sparsity for improving the estimation accuracy of the angle parameter. Unfortunately, whether the schemes with off-grid error [17,18,19,20,21] or the frameworks under unknown mutual coupling [26,27,28,29,30,31,32,33], they focus on only one factor that we are interested in, i.e., off-grid error or unknown mutual coupling.

In fact, there are few studies on off-grid DOA estimation under unknown mutual coupling [38,39,40]. According to [38], a revised Root-OGSBL (Root-SBL) method is reported for off-grid estimation with unknown mutual coupling. Moreover, a novel sparse Bayesian learning with mutual coupling (SBLMC) estimator [39] for MIMO radar is developed by the expectation maximum (EM) principle to iteratively update unknown parameters, such as noise variance, mutual coupling coefficients, and off-grid gap vector. Nevertheless, such estimation superiority is at the expense of computational burden. Subsequently, an off-grid sparse recovery algorithm under unknown mutual coupling (OGSRMC) [40] is presented by iteratively updating DOAs, unknown mutual coupling, and off-grid parameters, which is superior in computational complexity and inferior in estimation accuracy to the SBLMC method.

In this work, a reweighted off-grid SpSF (Re-OGSpSF) scheme is presented for DOA estimation in the environment of unknown mutual coupling. The proposed estimator not only incorporates the off-grid error term into the underlying on-grid sparse recovery model to enhance the robustness, but also utilizes the reweighted strategy to ensure accuracy. Hence, the proposed Re-OGSpSF framework can realize high-precision continuous DOA estimation with low computational burden by adopting a coarse grid interval. Massive experimental results are displayed to validate the above inferences. The main contributions of this paper are listed as follows:

(1) Designing a selection matrix to eliminate the unknown mutual coupling interference;
(2) Formulating an improved off-grid SpSF (OGSpSF) framework for off-grid DOA estimation by joint sparse recovery;
(3) Developing a MUSIC-like weighted matrix to reweight the OGSpSF scheme for strengthening the solution’s sparsity.

The remainder of this article is structured as follows: In Section 2, an actual data model affected by unknown mutual coupling is first defined. Then, a novel Re-OGSpSF scheme is explored for direction finding with unknown mutual coupling of the sensor array in Section 3. Subsequently, Section 4 presents the relevant remarks of this work. Afterwards, a series of simulation results are given to validate the superiority of the proposed framework in Section 5. Finally, Section 6 presents the conclusion of this paper.

Additionally, the relevant key notations involved in this work are explained in the following Table 1.

## 2. The Coupled Signal Model

Consider a uniform linear array (ULA) configured with *M* omnidirectional sensors, each separated by the spacing *d*. $d\leq \lambda_{d}/2$, where $\lambda_{d}$ refers to the signal wavelength. *K* narrowband uncorrelated sources ${\left\{s_{k}\right\}}_{k=1}^{K}$ are incident on the ULA from distinct directions ${\left\{\theta_{k}\right\}}_{k=1}^{K}$, where there is reason to believe that *K* is known exactly in advance [24,25]. Therefore, the ideal array output can be structured as

(1) x(t)=As(t)+n(t)

where $x\left(t\right)={\left[x_{1}\left(t\right),x_{2}\left(t\right),\ldots ,x_{M}\left(t\right)\right]}^{T}\in C^{M\times 1}$ means the ideal received data. $s\left(t\right)={\left[s_{1}\left(t\right),s_{2}\left(t\right),\ldots ,s_{K}\left(t\right)\right]}^{T}\in C^{K\times 1}$ reveals the signal vector. $n\left(t\right)={\left[n_{1}\left(t\right),n_{2}\left(t\right),\ldots ,n_{M}\left(t\right)\right]}^{T}\in C^{M\times 1}$ represents a stochastic Gaussian white noise vector, which follows $n\left(t\right)∼N\left(0,\sigma^{2}I_{M}\right)$ with noise power $\sigma^{2}$. $A=\left[a\left(\theta_{1}\right),a\left(\theta_{2}\right),\ldots ,a\left(\theta_{K}\right)\right]\in C^{M\times K}$ denotes the array manifold matrix, where $a\left(\theta_{k}\right)={\left[1,\eta \left(\theta_{k}\right),\ldots ,\eta^{M-1}\left(\theta_{k}\right)\right]}^{T}\in C^{M\times 1}$ refers to the steering vector with $\eta \left(\theta_{k}\right)=e^{-j2\pi d/\lambda_{d}\sin\theta_{k}}$.

For a fixed array, the greater the number of antennas, the closer the sensors, and the greater the possibility of space electromagnetic fields interaction between them. In other words, the sensors are too close to escape such interaction, causing unknown mutual coupling disturbance between closely-spaced sensors. Under such a scenario, the ideal structure of the array manifold is disturbed and then coupled as

(2) $$\hat{a}\left(\theta_{k}\right)=Ga\left(\theta_{k}\right)$$

where $G\in C^{M\times M}$ represents a mutual coupling matrix (MCM). According to [26], it is rational for MCM to depict its intrinsic properties with the complex banded symmetric Toeplitz structure. The mutual coupling coefficients between the sensors are inversely proportional to their spacing, i.e., the greater the antenna distance, the smaller the mutual coupling coefficients. What is more, the magnitude of the mutual coupling coefficient decreases rapidly when the antenna spacing increases [23]. In this way, it is reasonable to assume that the sensors far enough away are immune to the effect of unknown mutual coupling, i.e., the corresponding coefficients in the MCM are zero. Therefore, MCM is typically modeled as a banded symmetric Toeplitz matrix with just a few nonzero coefficients [23,29], i.e.,

(3) $$\begin{array}{cc} G & =\mathrm{Toeplitz}(\left[1,g_{1},\ldots ,g_{H},0_{1\times (M-H-1)}\right])\\ \; & ={\left[\begin{array}{cccccccccc} \;\;1\; & \;g_{1}\; & \;\cdots \; & \;g_{H}\; & \;\; & \;\; & \;\; & \;\; & \;\;\;\;\\ \;\;g_{1}\; & \;1\; & g_{1}\; & \;\cdots \; & \;g_{H}\; & \;\; & \;\; & \;0\; & \;\;\;\;\\ \;\;\vdots \; & \;\ddots \; & \;\ddots \; & \;\ddots \; & \;\ddots \; & \;\ddots \; & \;\; & \;\; & \;\; & \;\;\;\;\\ \;\;g_{H}\; & \;\cdots \; & \;g_{1}\; & \;1\; & \;g_{1}\; & \;\cdots \; & g_{H}\; & \;\; & \;\;\;\;\\ \;\;\; & \;\ddots \; & \;\vdots \; & \;\ddots \; & \;\ddots \; & \;\ddots \; & \;\ddots \; & \;\ddots \; & \;\;\;\\ \;\;\; & \;\; & \;g_{H}\; & \;\cdots \; & g_{1}\; & \;1\; & \;g_{1}\; & \;\cdots \; & \;g_{H}\;\;\;\\ \;\;\; & \;\; & \;\; & \;\ddots \; & \;\vdots \; & \;\ddots \; & \;\ddots \; & \;\ddots \; & \;\vdots \;\;\;\\ \;\;\; & \;0\; & \;\; & \;\; & \;g_{H}\; & \;\cdots \; & \;g_{1}\; & \;1\; & \;g_{1}\;\;\;\\ \;\;\; & \;\; & \;\; & \;\; & \;\; & \;g_{H}\; & \;\cdots \; & g_{1}\; & 1\;\;\; \end{array}\right]}_{M\times M} \end{array}$$

where ${\left\{g_{c}\right\}}_{c=1}^{H}$ reveal *H* unknown non-zero mutual coupling coefficients that satisfy $0<\left|g_{H}\right|<\left|g_{H-1}\right|<,\ldots ,<\left|g_{1}\right|<\left|g_{0}\right|=1$.

Under the condition of unknown mutual coupling, (1) should be rewritten as

(4) x(t)=GAs(t)+n(t)

By collecting *T* snapshots, the coupled array output matrix can be formed as

(5) X=GAS+N

where $X=\left[x\left(t_{1}\right),x\left(t_{1}\right),\ldots ,x\left(t_{T}\right)\right]\in C^{M\times T}$ refers to the coupled array output. $\hat{A}=\mathrm{GA}=\left[\hat{a}\left(\theta_{1}\right),\hat{a}\left(\theta_{2}\right),\ldots ,\hat{a}\left(\theta_{K}\right)\right]\in C^{M\times K}$ reveals the coupled array manifold matrix with $\hat{a}\left(\theta_{k}\right)=Ga\left(\theta_{k}\right)\left(K=1,2,\ldots ,K\right)$. $S=\left[s\left(t_{1}\right),s\left(t_{2}\right),\ldots ,s\left(t_{T}\right)\right]\in C^{K\times T}$ is the source matrix. $N=\left[n\left(t_{1}\right),n\left(t_{2}\right),\ldots ,n\left(t_{T}\right)\right]\in C^{M\times T}$ indicates the noise matrix.

## 3. Re-OGSpSF for DOA Estimation in Sensor Array with Unknown Mutual Coupling

In this section, an effective Re-OGSpSF estimator is structured for off-grid DOA estimation with sensor array under the condition of unknown mutual coupling. Under such a framework, an enhanced OGSpSF representation model is constructed by integrating the off-grid error term obtained from the first-order Taylor approximation of the higher-order term into the underlying on-grid sparse recovery one. What is more, a weighted matrix is achieved by the MUSIC-like principle to reweight the OGSpSF scheme for improving the estimation accuracy. Hence, such an off-grid estimator can provide continuous DOA estimation to enforce the estimation accuracy or/and reduce the computational burden when an off-grid case occurs. In this way, it enables effective and efficient high-precision continuous off-grid estimation that would be more suitable for real-time direction finding.

### 3.1. Eliminating the Effect of Unknown Mutual Coupling

Obviously, the actual steering vector contains unknown mutual coupling coefficients, indicating that numerous DOA estimation approaches, including the SSR manner, will fail to work. In order to study the DOA estimation issue from the SSR perspective, it is quite necessary for the sparse reconstruction model to structure the effective over-complete dictionary. In other words, the unknown mutual coupling interference should be removed first for direction finding. According to (3), the specific selection matrix [29] is designed as

(6) $$J=[0_{(M-2H)\times H},I_{\left(M-2H\right)},0_{(M-2H)\times H}]$$

Then, multiplying the selection matrix J by the coupled steering vector in (2) yields

(7) $$\tilde{a}\left(\theta_{k}\right)=J\hat{a}\left(\theta_{k}\right)=JGa\left(\theta_{k}\right)=\xi_{k}\vec{a}\left(\theta_{k}\right)$$

where $\xi_{k}=\sum_{h=-H}^{H}g_{\left|h\right|}e^{-j2\pi d/\lambda_{d}\left(h+H\right)\sin\theta_{k}}$ indicates a constant for each true target. $\vec{a}\left(\theta_{k}\right)={\left[1,\eta \left(\theta_{k}\right),\ldots ,\eta^{\vec{M}-1}\left(\theta_{k}\right)\right]}^{T}\in C^{\vec{M}\times 1}$ refers to a new array manifold with $\vec{M}=M-2H$. It successfully decouples the DOAs parameter from the unknown mutual coupling coefficients, despite not using H antennas on each side of the ULA.

Inspired by (7), (5) can be greatly decoupled as

(8) $$\begin{array}{c} Y=JX=J\left(GAS+N\right)=JGAS+JN=\vec{A}\Lambda S+\vec{N}=\vec{A}\vec{S}+\vec{N} \end{array}$$

where $Y=\left[y\left(t_{1}\right),y\left(t_{1}\right),\ldots ,y\left(t_{T}\right)\right]\in C^{\vec{M}\times T}$ reveals the array output free from unknown mutual coupling interference. $\vec{A}=\left[\vec{a}\left(\theta_{1}\right),\vec{a}\left(\theta_{2}\right),\ldots ,\vec{a}\left(\theta_{K}\right)\right]\in C^{\vec{M}\times K}$ denotes the decoupled array manifold matrix, like A in (1). $\Lambda =\mathrm{diag}\left\{\xi_{1},\xi_{2},\ldots ,\xi_{K}\right\}\in C^{K\times K}$ stands for a diagonal matrix that can be integrated with matrix S to generate a revised signal matrix $\vec{S}$. $\vec{N}\in C^{\vec{M}\times T}$ represents the modified noise matrix.

### 3.2. SpSF Principle

According to (8), the covariance matrix of Y can be denoted as

(9) $$\begin{array}{c} R=E\{YY^{H}\}=\vec{A}R_{\vec{S}}{\vec{A}}^{H}+R_{\vec{N}}=\vec{A}\mathrm{diag}\left\{\rho_{1}^{2},\rho_{2}^{2},\ldots ,\rho_{K}^{2}\right\}{\vec{A}}^{H}+\sigma^{2}I_{\vec{M}} \end{array}$$

where $R_{\vec{N}}=\sigma^{2}I_{\vec{M}}$ denotes the noise covariance matrix. $R_{\vec{S}}=\mathrm{diag}\left\{\rho_{1}^{2},\rho_{2}^{2},\ldots ,\rho_{K}^{2}\right\}$ means the signal covariance matrix, where $\rho_{k}^{2}$ stands for the *k*-th signal power.

Based on (9), it can be found that the array aperture can be enlarged by performing the covariance vectorization operation. Then, vectoring R yields

(10) $$r_{v}=\mathrm{vec}\left(R\right)=\left({\vec{A}}^{*}⊙\vec{A}\right)\vec{u}+{\vec{n}}_{v}={\vec{A}}_{v}\vec{u}+{\vec{n}}_{v}$$

where ${\vec{A}}_{v}={\vec{A}}^{*}⊙\vec{A}=\left[{\vec{a}}_{v}\left(\theta_{1}\right),{\vec{a}}_{v}\left(\theta_{2}\right),\ldots ,{\vec{a}}_{v}\left(\theta_{K}\right)\right]\in C^{{\vec{M}}^{2}\times K}$ is the virtual array manifold matrix, where ${\vec{a}}_{v}\left(\theta_{k}\right)={\vec{a}}^{*}\left(\theta_{k}\right)⊗\vec{a}\left(\theta_{k}\right)\in C^{{\vec{M}}^{2}\times 1}$ denotes the virtual array manifold, unlike the steering vector $\vec{a}\left(\theta_{k}\right)$ in (8). Obviously, such a vectorized signal model effectively increases the degrees of freedom (DOFs) of the virtual array and expands the array aperture. $\vec{u}={\left[\rho_{1}^{2},\rho_{2}^{2},\ldots ,\rho_{K}^{2}\right]}^{T}\in C^{K\times 1}$ and ${\vec{n}}_{v}=\mathrm{vec}\left(R_{\vec{N}}\right)=\sigma^{2}\mathrm{vec}\left(I_{\vec{M}}\right)\in C^{{\vec{M}}^{2}\times 1}$.

Inspired by (10), a sparsity-inducing scheme can be structured for DOA estimation. Through discretizing the spatial domain uniformly and densely enough, a set of candidate directions can be achieved, that is, $\bar{\theta }=\left\{{\bar{\theta }}_{1},{\bar{\theta }}_{2},\ldots ,{\bar{\theta }}_{N}\right\}\left(N⩾K\right)$. In this way, it is reasonable to assume that all source DOAs fall exactly on the candidate directions of set $\bar{\theta }$. Then, an over-complete dictionary can be modeled as

(11) $$\begin{array}{c} {\bar{A}}_{v}\;=\;{\bar{A}}^{*}⊙\bar{A}\;=\;\left[{\vec{a}}_{v}\left({\bar{\theta }}_{1}\right),{\vec{a}}_{v}\left({\bar{\theta }}_{2}\right),\ldots ,{\vec{a}}_{v}\left({\bar{\theta }}_{N}\right)\right]\;\in C^{{\vec{M}}^{2}\times N} \end{array}$$

where $\bar{A}=\left[\vec{a}\left({\bar{\theta }}_{1}\right),\vec{a}\left({\bar{\theta }}_{2}\right),\ldots ,\vec{a}\left({\bar{\theta }}_{N}\right)\right]\in C^{\vec{M}\times N}$ and ${\vec{a}}_{v}\left({\bar{\theta }}_{n}\right)={\vec{a}}^{*}\left({\bar{\theta }}_{n}\right)⊗\vec{a}\left({\bar{\theta }}_{n}\right)\in C^{{\vec{M}}^{2}\times 1}$.

Combining (11), (10) can be sparsely expressed as

(12) $$r_{v}=\left({\bar{A}}^{*}⊙\bar{A}\right)\bar{u}+{\vec{n}}_{v}={\bar{A}}_{v}\bar{u}+{\vec{n}}_{v}$$

where $\bar{u}={\left[{\bar{\rho }}_{1}^{2},{\bar{\rho }}_{2}^{2},\ldots ,{\bar{\rho }}_{N}^{2}\right]}^{T}\in C^{N\times 1}$ reveals the *K*-sparse vector of signal power due to the existence of *K* sources. *K* non-zero entries in $\bar{u}$ correspond to the desired DOAs and are equal to that in $\vec{u}$, i.e., $\{{\rho_{k}^{2}\}}_{k=1}^{K}$. In this way, such a direction-finding problem can be turned into a sparse reconstruction issue, where DOAs can be determined by scanning the locations of *K* non-zero entries in the sparse vector $\bar{u}$.

To the best of our knowledge, the $l_{0}$-norm penalty is considered to be the theoretically optimal choice for measuring sparsity. Unfortunately, as a classical non-convex and non-deterministic polynomial (NP)-hard issue, $l_{0}$-norm penalty is mathematically intractable. That is to say, $l_{0}$-norm penalty may not be applicable in actual direction finding. Inspired by [22,33], it is rational for the sparsity-inducing scheme to recover the sparse matrix via using $l_{1}$-norm optimization rather than $l_{0}$-norm minimization. Through convex approximation, $l_{1}$-norm penalty turns the non-convex scenario into a convex one to relieve the computational load. Following this thought, the $l_{1}$-norm penalty scheme can be structured as

(13) $$\min∥\bar{u}{∥}_{1}\;\;\;s.t.\;\;\;∥r_{v}-{\bar{A}}_{v}\bar{u}{∥}_{2}^{2}\;⩽\;\varsigma ,\;\;\;\bar{u}\;⩾\;0$$

where ς refers to the regularization parameter, which balances the fitting error and signal sparsity and is crucial to robust sparse recovery. Its detailed explanation can be found in Remark 1 of the Related Remarks in Section 4.

In (9), R is an ideal covariance matrix based on the infinite number of snapshots, which is unavailable in practice. In actual situations, R is usually replaced by the sample covariance matrix under a finite number of snapshots *T*, i.e.,

(14) $$\bar{R}=\frac{1}{T}\sum_{t=1}^{T}Y\left(t\right)Y^{H}\left(t\right)$$

It is easy to find that there is a fitting error between R and $\bar{R}$ caused by the finite snapshots, that is, $\Delta R=\bar{R}-R$. In this way, (10) should be revised as follows:

(15) $${\bar{r}}_{v}=\mathrm{vec}\left(\bar{R}\right)=\mathrm{vec}\left(R+\Delta R\right)=r_{v}+\Delta r={\vec{A}}_{v}\vec{u}+{\vec{n}}_{v}+\Delta r$$

where $\Delta r={\bar{r}}_{v}-r_{v}=\mathrm{vec}\left(\Delta R\right)=\mathrm{vec}\left(\bar{R}-R\right)$ refers to the covariance fitting error vector.

Then, (15) can be further sparsely modeled as

(16) $${\bar{r}}_{v}=\left({\bar{A}}^{*}⊙\bar{A}\right)\bar{u}+{\vec{n}}_{v}+\Delta r={\bar{A}}_{v}\bar{u}+{\vec{n}}_{v}+\Delta r$$

Inspired by [10,11], Δr obeys the distribution as follows:

(17) $$\Delta r∼\mathrm{AsN}(0_{{\vec{M}}^{2}\times 1},W)$$

where $W=\frac{R^{T}⊗R}{T}$. AsN(μ,ϖ) indicates the asymptotically normal (AsN) distribution, whose mean and variance are equal to μ and ϖ, respectively. Obviously, Δr does not obey the standard normal distribution and ς is hard to calculate at this time. Whereas, with the help of asymptotic characteristic of Δr, it is easy to determine the parameter ς.

Combining the principle of linear algebra yields

(18) $$W^{-\frac{1}{2}}\Delta r∼\mathrm{AsN}\left(0_{{\vec{M}}^{2}\times 1},I_{{\vec{M}}^{2}}\right)$$

As depicted in (18), it can be directly deduced that

(19) $${∥W^{-\frac{1}{2}}\Delta r∥}_{2}^{2}∼\mathrm{As}\chi^{2}\left({\vec{M}}^{2}\right)$$

where $\mathrm{As}\chi^{2}\left(\nu \right)$ obeys the asymptotically chi-square distribution with ν DOFs.

Let $\bar{W}=\frac{{\bar{R}}^{T}⊗\bar{R}}{T}$ and ${\bar{n}}_{v}={\bar{\sigma }}^{2}\mathrm{vec}\left(I_{\vec{M}}\right)$ be estimates of W and ${\vec{n}}_{v}$, respectively. Additionally, ${\bar{\sigma }}^{2}$ is the estimated noise power. Combining (16) with (19) yields

(20) $$\min{∥\bar{u}∥}_{1}\;\;\;s.t.\;\;\;{∥{\bar{W}}^{-\frac{1}{2}}\left({\bar{r}}_{v}-{\bar{n}}_{v}-{\bar{A}}_{v}\bar{u}\right)∥}_{2}\;⩽\;\sqrt{\varsigma },\;\;\bar{u}\;⩾\;0$$

In this way, DOA estimation can be achieved by the spatial spectrum of the recovered sparse vector $\bar{u}$.

### 3.3. Off-Grid SpSF (OGSpSF) for DOA Estimation

It is clear that the sparse vector $\bar{u}$ in (20) can indeed be recovered for DOA estimation. Unfortunately, it only considers the on-grid case by default. In practice, the spatial domain can hardly be discretized adequately to generate continuous candidate sampling grids, but the target directions are continuous variables. Thus, it is difficult for the predefined grid points to accurately match the actual angles. In other words, there is an inevitable off-grid gap between the desired DOAs and the candidate grid points, degrading the estimation performance to some extent. Inspired by the linear approximation thought in [17], an effective OGSpSF representation model is formed by integrating the off-grid error term derived from the first-order Taylor approximation into the original on-grid sparse recovery one to guarantee the robustness and accuracy.

It is known that the candidate direction ${\bar{\theta }}_{n}$ is mismatched with the true DOA $\theta_{K}$ under the off-grid scenario. Furthermore, it is not hard to find that each element of the virtual array manifold ${\vec{a}}_{v}\left(\theta_{k}\right)$ in (10) can be represented as ${\left[{\vec{a}}_{v}\left(\theta_{k}\right)\right]}_{\vec{m}}=e^{-j2\pi d/\lambda_{d}\vec{m}\sin\theta_{k}}$ with integer $\vec{m}\in \left[1-\vec{M},\vec{M}-1\right]$. Resorting to ${\left[{\vec{a}}_{v}\left({\bar{\theta }}_{n}\right)\right]}_{\vec{m}}$ in (11), ${\left[{\vec{a}}_{v}\left(\theta_{k}\right)\right]}_{\vec{m}}$ can be depicted as

(21) $${\left[{\vec{a}}_{v}\left(\theta_{k}\right)\right]}_{\vec{m}}=e^{-j2\pi d/\lambda_{d}\vec{m}\delta_{n}}\times {\left[{\vec{a}}_{v}\left({\bar{\theta }}_{n}\right)\right]}_{\vec{m}}$$

where ${\left[{\vec{a}}_{v}\left({\bar{\theta }}_{n}\right)\right]}_{\vec{m}}=e^{-j2\pi d/\lambda_{d}\vec{m}\sin{\bar{\theta }}_{n}}$. ${\bar{\theta }}_{n}\in \bar{\theta }$ is the sampling grid point closest to $\theta_{k}$ and $\delta_{n}=\sin\theta_{k}-\sin{\bar{\theta }}_{n}$ indicates the offset parameter. Inspired by the Taylor expansion in algebraic theory, $e^{-j2\pi d/\lambda_{d}\vec{m}\delta_{n}}$ can be replaced by its first-order Taylor approximation, i.e., $e^{-j2\pi d/\lambda_{d}\vec{m}\delta_{n}}\approx \left[1+\left(-j2\pi d/\lambda_{d}\vec{m}\delta_{n}\right)\right]$.

Then, (21) can be approximated to

(22) $$\begin{array}{cc} {}_{\vec{m}} & =e^{-j2\pi d/\lambda_{d}\vec{m}\delta_{n}}\times {\left[{\vec{a}}_{v}\left({\bar{\theta }}_{n}\right)\right]}_{\vec{m}}\\ \; & \approx \left[1+\left(-j2\pi d/\lambda_{d}\vec{m}\delta_{n}\right)\right]{\left[{\vec{a}}_{v}\left({\bar{\theta }}_{n}\right)\right]}_{\vec{m}}\\ \; & \approx {\left[{\vec{a}}_{v}\left({\bar{\theta }}_{n}\right)\right]}_{\vec{m}}+\left(-j2\pi d/\lambda_{d}\vec{m}\right){\left[{\vec{a}}_{v}\left({\bar{\theta }}_{n}\right)\right]}_{\vec{m}}\delta_{n} \end{array}$$

According to (22), it can be deduced that the virtual array manifold ${\vec{a}}_{v}\left(\theta_{k}\right)$ can be composed of ${\vec{a}}_{v}\left({\bar{\theta }}_{n}\right)$ and ${\vec{b}}_{v}\left({\bar{\theta }}_{n}\right)\delta_{n}$, where ${\vec{b}}_{v}\left({\bar{\theta }}_{n}\right)$ can be recorded as

(23) $${\vec{b}}_{v}\left({\bar{\theta }}_{n}\right)=\Gamma {\vec{a}}_{v}\left({\bar{\theta }}_{n}\right)$$

where $\Gamma =\mathrm{diag}\left\{\phi \right\}\in C^{{\vec{M}}^{2}\times {\vec{M}}^{2}}$ indicates a diagonal matrix with $\phi =\left[\phi_{1},\phi_{2},\ldots ,\phi_{{\vec{M}}^{2}}\right]=-j2\pi d/\lambda_{d}[0,1,\ldots ,\vec{M}-1,-1,0,\ldots ,\vec{M}-2,\ldots ,2-\vec{M},3-\vec{M},\ldots ,1,1-\vec{M},2-\vec{M},\ldots ,0]\in C^{1\times {\vec{M}}^{2}}$. Obviously, its diagonal element corresponding to ${\left[{\vec{a}}_{v}\left({\bar{\theta }}_{n}\right)\right]}_{\vec{m}}$ is equal to $-j2\pi d/\lambda_{d}\vec{m}$. By separating the unknown variable $\delta_{n}$ and the known parameter $-j2\pi d/\lambda_{d}\vec{m}$, ${\vec{b}}_{v}\left({\bar{\theta }}_{n}\right)$ can finally be known, despite $\delta_{n}$ being hard to determine due to the presence of $\sin\theta_{k}$.

In this way, another over-complete dictionary ${\bar{B}}_{v}$, except for ${\bar{A}}_{v}$ in (11), can be further structured to help construct the off-grid sparse recovery model, which takes the following form:

(24) $${\bar{B}}_{v}=\Gamma {\bar{A}}_{v}=\left[{\vec{b}}_{v}\left({\bar{\theta }}_{1}\right),{\vec{b}}_{v}\left({\bar{\theta }}_{2}\right),\ldots ,{\vec{b}}_{v}\left({\bar{\theta }}_{N}\right)\right]\in C^{{\vec{M}}^{2}\times N}$$

Following (22) and (24), the on-grid sparse representation model in (16) should be refined to an OGSpSF recovery one, i.e.,

(25) $${\bar{r}}_{v}=\mathrm{vec}\left(\bar{R}\right)=\left({\bar{A}}_{v}+\Gamma {\bar{A}}_{v}\Delta \right)\bar{u}+{\vec{n}}_{v}+\Delta r={\bar{A}}_{v}\bar{u}+{\bar{B}}_{v}\bar{v}+{\vec{n}}_{v}+\Delta r$$

where $\Delta =\mathrm{diag}\left\{\delta_{1},\delta_{2},\ldots ,\delta_{N}\right\}\in C^{N\times N}$ denotes a diagonal matrix associated with the unknown DOAs information that can be integrated with $\bar{u}$ to structure a new virtual signal power vector $\bar{v}$, i.e., $\bar{v}=\Delta \bar{u}={\left[{\bar{q}}_{1}^{2},{\bar{q}}_{2}^{2},\ldots ,{\bar{q}}_{N}^{2}\right]}^{T}={\left[\delta_{1}{\bar{\rho }}_{1}^{2},\delta_{2}{\bar{\rho }}_{2}^{2},\ldots ,\delta_{N}{\bar{\rho }}_{N}^{2}\right]}^{T}\in C^{N\times 1}$.

On the one hand, it is preferable for the estimated spatial spectrum to associate the non-zero signal powers with the nearest sampling grid points. ${\bar{\rho }}_{n}^{2}$ in the sparse vector $\bar{u}$ means the signal power centered at the sampling grid point ${\bar{\theta }}_{n}$. Thus, the following restriction is given as:

(26) $$\gamma_{n}⩽\delta_{n}⩽\beta_{n},n=1,2,\ldots ,N$$

where

(27) $$\gamma_{n}=\sin\frac{{\bar{\theta }}_{n-1}+{\bar{\theta }}_{n}}{2}-\sin{\bar{\theta }}_{n},n=2,3,\ldots ,N$$

(28) $$\beta_{n}=\sin\frac{{\bar{\theta }}_{n+1}+{\bar{\theta }}_{n}}{2}-\sin{\bar{\theta }}_{n},n=1,2,\ldots ,N-1$$

where $\gamma_{1}=0$ and $\beta_{N}=0$.

On the other hand, as $\bar{u}⩾0$ (i.e., ${\bar{\rho }}_{n}^{2}⩾0,n=1,2,\ldots ,N$), the following constraint can be further deduced from (26):

(29) $$\gamma_{n}{\bar{\rho }}_{n}^{2}⩽{\bar{q}}_{n}^{2}⩽\beta_{n}{\bar{\rho }}_{n}^{2},n=1,2,\ldots ,N$$

Observing (25), when there is no source from the candidate directions set centered on ${\bar{\theta }}_{n}$, the corresponding spatial spectrum entries in the sparse vectors $\bar{u}$ and $\bar{v}$ are ${\bar{u}}_{n}=0$ and ${\bar{v}}_{n}=0$, respectively. Hence, $\bar{u}$ takes the same support set (i.e., row sparsity) as $\bar{v}$, which promotes group sparsity between the sparse vectors $\bar{u}$ and $\bar{v}$ [17]. This group sparsity of $\left[\bar{u},\bar{v}\right]$ can be exploited as the objective function, and then an OGSpSF scheme can be built for off-grid DOA estimation, i.e.,

(30) $$\begin{array}{cc} \min & {∥P^{l_{2}}∥}_{1}\;\;\;\;s.t.\;\;\;\;{∥{\bar{W}}^{-\frac{1}{2}}\left({\bar{r}}_{v}-{\bar{n}}_{v}-{\bar{A}}_{v}\bar{u}-{\bar{B}}_{v}\bar{v}\right)∥}_{2}⩽\sqrt{\varsigma }\\ \; & \;\;\;\;\gamma_{n}{\bar{\rho }}_{n}^{2}⩽{\bar{q}}_{n}^{2}⩽\beta_{n}{\bar{\rho }}_{n}^{2},\;\;{\bar{\rho }}_{n}^{2}⩾0,\;\;n=1,2,\ldots ,N \end{array}$$

where $P^{l_{2}}={\left[\bar{u},\bar{v}\right]}^{l_{2}}={\left[{\bar{p}}_{1},{\bar{p}}_{2},\ldots ,{\bar{p}}_{N}\right]}^{T}\in C^{N\times 1}$, whose *n*th entry ${\bar{p}}_{n}$ equals the $l_{2}$-norm of the *n*th row in $P=[\bar{u},\bar{v}]$, i.e.,

(31) $${\bar{p}}_{n}=\sqrt{\sum_{\bar{j}=1}^{2}{\left(p_{n,\bar{j}}\right)}^{2}},\;\;n=1,2,\ldots ,N$$

where $p_{n,\bar{j}}$ is an element in P with the coordinate index $\left(n,\bar{j}\right)$.

Obviously, the off-grid estimation trouble was finally transformed into a joint sparse recovery problem by exploiting the group sparsity of $P=[\bar{u},\bar{v}]$. With the help of the recovered sparse vectors $\bar{u}$ and $\bar{v}$ in (30), the problem of off-grid DOAs can be solved.

Specifically, the positions of *K* peaks, i.e., $n_{1},n_{2},\ldots ,n_{K}$, can first be determined by plotting the spatial spectrum of the recovered sparse vector $\bar{u}$. Subsequently, the final off-grid DOA estimation can be computed based on the recovered sparse vectors $\bar{u}$ and $\bar{v}$, as follows:

(32) $${\hat{\theta }}_{k}=\arcsin\left[\sin{\bar{\theta }}_{n_{k}}+{\bar{v}}_{n_{k}}/{\bar{u}}_{n_{k}}\right],\;\;k=1,2,\ldots ,K$$

where ${\bar{u}}_{n_{k}}>0,k=1,2,\ldots ,K$ is supposed here.

Such a convex constraint framework reasonably utilizes the group sparsity of $P=[\bar{u},\bar{v}]$ to facilitate continuous off-grid DOA estimation. Consequently, it is favorable for direction finding under off-grid condition to enhance the robustness and accuracy. What is more, this framework can quickly achieve high-precision continuous DOA estimation by exploiting a coarse grid interval to reduce the computational burden. In this way, such feasible off-grid scheme is applicable to real-time direction finding scenarios.

Unfortunately, the penalty scheme in (30) realizes off-grid DOA estimation by relaxing the $l_{0}$-norm constraint to the $l_{1}$-norm one, generating an approximation error and compromising the recovery accuracy. In view of the limited recovery performance, the following subsection will reweight the OGSpSF framework in (30) to enhance accuracy.

### 3.4. Reweighted OGSpSF (Re-OGSpSF) for DOA Estimation in Sensor Array with Unknown Mutual Coupling

As the $l_{1}$-norm is just a convex approximation of the $l_{0}$-norm, there will inevitably be a difference between these two penalty ways. Different from the impartial $l_{0}$-norm optimization, the penalty for larger coefficients outweighs that for smaller coefficients in the $l_{1}$-norm constraint scheme, which means that the two sparse vectors $\bar{u}$ and $\bar{v}$ or the sparse vector $P^{l_{2}}$ in (30) cannot be recovered well. For acquiring better recovery performance in the $l_{1}$-norm constraint scheme, a weighted attempt achieved by the MUSIC-like principle [22,33] is carried out to strengthen the solution’s sparsity.

First, imposing eigenvalue decomposition on $\bar{R}$ yields

(33) $$\bar{R}=\sum_{\vec{m}=1}^{\vec{M}}\tau_{\vec{m}}\alpha_{\vec{m}}\alpha_{\vec{m}}^{H}=E_{s}\Lambda_{s}E_{s}^{H}+E_{n}\Lambda_{n}E_{n}^{H}$$

where $\tau_{\vec{m}}$ and $\alpha_{\vec{m}}$ stand for the $\vec{m}$th eigenvalue and the corresponding eigenvector of the sample covariance matrix $\bar{R}$, respectively. $\Lambda_{s}=\mathrm{diag}\left\{\tau_{1},\tau_{2},\ldots ,\tau_{K}\right\}\in C^{K\times K}$ and $\Lambda_{n}=\mathrm{diag}\left\{\tau_{K+1},\tau_{K+2},\ldots ,\tau_{\vec{M}}\right\}\in C^{\left(\vec{M}-K\right)\times \left(\vec{M}-K\right)}$. The signal subspace $E_{s}$ and the noise subspace $E_{n}$ are formed by eigenvectors corresponding to *K* larger eigenvalues and $\vec{M}-K$ smaller eigenvalues, respectively.

Then, as the decoupled steering vector is orthogonal to its noise subspace, a spatial spectrum function of MUSIC-like is structured as

(34) $$f_{\mathrm{MUSIC}}\left(\theta \right)=\arg\min\left\{\det\{{\vec{a}}^{H}\left(\theta \right)E_{n}E_{n}^{H}\vec{a}\left(\theta \right)\}\right\}$$

Since the over-complete dictionary ${\bar{A}}_{v}$ in (11) is obtained by sparsely representing ${\vec{A}}_{v}$ in (10), it is still orthogonal to its noise subspace, facilitating the weights establishment. Without loss of generality, the over-complete dictionary ${\bar{A}}_{v}$ can be partitioned into two sub-matrices, i.e., ${\bar{A}}_{v}=\left[{\bar{A}}_{v_{1}},{\bar{A}}_{v_{2}}\right]$. The former, ${\bar{A}}_{v_{1}}$, is assumed to be formed by *K* array manifolds corresponding to the desired target directions, while the latter, ${\bar{A}}_{v_{2}}$, is thought to be made up of the remaining N−K steering vectors. According to (34), the initial weights can be depicted as

(35) $${\hat{z}}_{n}\;=\;\det\left\{{\vec{a}}^{H}\left({\bar{\theta }}_{n}\right)E_{n}E_{n}^{H}\vec{a}\left({\bar{\theta }}_{n}\right)\right\},\;\;n\;=\;1,2,\ldots ,N$$

where the initial weight ${\hat{z}}_{n}$ corresponds to the possible target angle ${\bar{\theta }}_{n}$. Based on (35), the weights can be further expressed as

(36) $${\bar{z}}_{n}={\hat{z}}_{n}/\max\left\{{\hat{z}}_{1},{\hat{z}}_{2},\ldots ,{\hat{z}}_{N}\right\},\;\;n\;=\;1,2,\ldots ,N$$

At last, a robust MUSIC-like weighted matrix achieved by (36) can be established as

(37) Z=diag{z}

where $z=\left[z_{1},z_{2}\right]=\left[{\bar{z}}_{1},{\bar{z}}_{2},\ldots ,{\bar{z}}_{N}\right]$. $z_{1}$ contains *K* weights corresponding to the real DOAs, smaller than that in $z_{2}$ formed by the residual weights. In particular, the weights in $z_{1}$ satisfy $z_{1}\to 0$ when the number of snapshots T→∞. Through applying the weighted matrix to the $l_{1}$-norm penalty framework, smaller weights in $z_{1}$ protect larger coefficients, while larger weights in $z_{2}$ punish smaller coefficients that are more likely to be zero. Accordingly, they can be punished as fairly as possible, no matter the larger or smaller coefficients in the sparse vectors $\bar{u}$ and $\bar{v}$.

Implanting the weighted matrix Z into the sparsity-inducing framework in (30) yields

(38) $$\begin{array}{cc} \min & {∥ZP^{l_{2}}∥}_{1}\;\;\;\;s.t.\;\;\;\;{∥{\bar{W}}^{-\frac{1}{2}}\left({\bar{r}}_{v}-{\bar{n}}_{v}-{\bar{A}}_{v}\bar{u}-{\bar{B}}_{v}\bar{v}\right)∥}_{2}⩽\sqrt{\varsigma }\\ \; & \;\;\;\;\gamma_{n}{\bar{\rho }}_{n}^{2}⩽{\bar{q}}_{n}^{2}⩽\beta_{n}{\bar{\rho }}_{n}^{2},\;\;{\bar{\rho }}_{n}^{2}⩾0,\;\;n=1,2,\ldots ,N \end{array}$$

The reweighted off-grid constraint framework in (38) can be successfully computed by using second order cone (SOC) programming software packages in MATLAB, such as CVX and Sedumi. Similarly, the final off-grid estimation can be realized by calculating (32).

So far, an efficient Re-OGSpSF approach has been developed to solve the off-grid DOA estimation problem in the environment of unknown mutual coupling. The entire procedure of the proposed method is given in Algorithm 1.
**Algorithm 1** Re-OGSpSF for DOA estimation in sensor array with unknown mutual coupling1:**Input:** The coupled received data X in (5);2:Formulate a decoupled array output Y using (8) by left multiplying the selection matrix J in (6) to eliminate the unknown mutual coupling effect;3:Compute the sample covariance matrix $\bar{R}$ of Y based on (14);4:Perform vectorization attempt on $\bar{R}$ to establish a vector data model given in (15);5:Impose eigenvalue decomposition on $\bar{R}$ by (33) to obtain the noise subspace $E_{n}$;6:Structure the over-complete dictionaries ${\bar{A}}_{v}$ in (11) and ${\bar{B}}_{v}$ in (24) to achieve an enhanced OGSpSF recovery model in (25);7:Design a MUSIC-like weighted matrix Z using (37) to reinforce the solution’s sparsity;8:Develop an effective Re-OGSpSF framework adopting (38) for off-grid DOA estimation by joint sparse recovery;9:**Output:** The recovered sparse vectors $\bar{u}$ and $\bar{v}$;10:Perform a 1-D spectrum search on the vector $\bar{u}$ to determine the indices of its *K* peaks.11:Calculate off-grid DOAs based on the recovered sparse vectors $\bar{u}$ and $\bar{v}$ by (32).

## 4. Related Remarks

**Remark** **1.** *To the best of our knowledge, it is extremely critical for (38) to determine the estimated noise power ${\bar{\sigma }}^{2}$ and an appropriate regularization parameter ς. On the one hand, ${\bar{\sigma }}^{2}$ can be computed via averaging $\vec{M}-K$ smaller eigenvalues of the sample covariance matrix $\bar{R}$. On the other hand, ς plays an extremely vital role in robust sparse reconstruction, balancing the fitting error and signal sparsity. Inspired by (19), the fitting error in (20), (30), and (38) follows the asymptotic chi-square distribution with ${\vec{M}}^{2}$ DOFs. Hence, ς can be determined via the upper bound of the fitting error with a high probability $\vec{\rho }$, i.e.,*

(39) $$\mathrm{Pr}\left\{\chi^{2}\left({\vec{M}}^{2}\right)\;⩽\;\varsigma \right\}=\vec{\rho },\;\;\;\varsigma =\chi_{\vec{\rho }}^{2}\left({\vec{M}}^{2}\right)$$

*where Pr{·} reveals the probability distribution of the event. In this way, ς can finally be determined by using the function $\mathrm{chi}2\mathrm{inv}(\vec{\rho },{\vec{M}}^{2})$ in MATLAB, where $\vec{\rho }=0.999$ is enough in this paper.*

**Remark** **2.** *This paper mainly focuses on off-grid DOA estimation under the condition of unknown mutual coupling, and then proposes an enhanced Re-OGSpSF algorithm. For the off-grid error, the off-grid representation is carried out in this article. To be specific, the off-grid error term obtained from the first-order Taylor approximation is integrated into the potential on-grid sparse model to construct the OGSpSF recovery model. To better and more intuitively verify the robustness of the proposed methodology to the off-grid error, a potential reweighted approach using the SpSF principle under the condition of unknown mutual coupling can be obtained by removing the off-grid representation in (21). To distinguish it from the proposed (Re-OGSpSF) framework, it can be defined as the reweighted SpSF (Re-SpSF) method. Specifically, according to the SpSF principle in Section 3.2, DOA estimation can be obtained with the help of the recovered sparse vector $\bar{u}$. However, the approximate penalty for replacing the $l_{0}$-norm constraint with the $l_{1}$-norm one causes limited reconstruction performance. Thus, the SpSF principle in Section 3.2 is combined with the weighted measure in Section 3.4 to form the Re-SpSF framework, i.e.,*

(40) $$\min{∥Z\bar{u}∥}_{1}\;\;\;s.t.\;\;\;{∥{\bar{W}}^{-\frac{1}{2}}\left({\bar{r}}_{v}-{\bar{n}}_{v}-{\bar{A}}_{v}\bar{u}\right)∥}_{2}\;⩽\;\sqrt{\varsigma },\;\;\bar{u}\;⩾\;0$$

*Due to the off-grid representation, the proposed scheme can provide continuous DOA estimation. From the perspective of estimation accuracy, the proposed Re-OGSpSF method mitigates the interference of off-grid error to a certain extent, enhances the robustness against off-grid error, and improves the estimation accuracy. In terms of angle estimation speed, the proposed Re-OGSpSF method can quickly perform high-precision continuous direction finding by setting a coarse grid interval, which relieves the computational burden and speeds up the DOA estimation. In general, the proposed methodology is effective and efficient in achieving high-precision continuous off-grid DOA estimation, which is more suitable for real-time direction finding in practical applications. This will be demonstrated in the subsequent simulation experiments.*

## 5. Numerical Simulation Results

In this section, extensive simulation experiments are performed to validate the estimation performance of the proposed Re-OGSpSF scheme. In order to show the effectiveness and efficiency, several estimators are simultaneously tested to compare with the proposed approach, including the $l_{1}$-SVD algorithm (recorded as SVD method) in [29], the BSR approach in [30], the SRACV method in [31], the reweighted SRACV framework (recorded as ReSRACV method) in [32], the potential Re-SpSF scheme (recorded as Re-SpSF method) in (40), and the Root-SBL estimator in [38]. Furthermore, as a benchmark for performance evaluation, the Cramer–Rao bound (CRB) in [41] is calculated as well. Additionally, the root means square error (RMSE) is computed to measure their accuracy, denoted as

(41) $$\mathrm{RMSE}=\sqrt{\frac{1}{LK}\sum_{l=1}^{L}\sum_{k=1}^{K}{\left(\theta_{l,k}-\theta_{k}\right)}^{2}}$$

where $\theta_{l,k}$ reveals the estimated value of the desired DOA $\theta_{k}$ at the *l*th Monte Carlo running. L=200 stands for the total number of Monte Carlo trials in this article.

In what follows, M=10 sensors are configured to structure a ULA, which are evenly separated by half-wavelength spacing, i.e., $d=\lambda_{d}/2$. Suppose there are K=2 narrow-band uncorrelated targets from distinct directions incident on the ULA, where DOAs are recorded as $\theta_{1}=-7.3^{\circ }$ and $\theta_{2}=4.2^{\circ }$, respectively. Moreover, there are three non-zero coefficients in the MCM with H=2, that is, $g_{0}=1$, $g_{1}=0.6864-j0.4776$, and $g_{2}=0.2069-j0.1024$. In addition, the discrete grid spacing ϵ of the spatial domain from $-90^{\circ }$ to $90^{\circ }$ is set to $1^{\circ }$.

**Figure 1:**
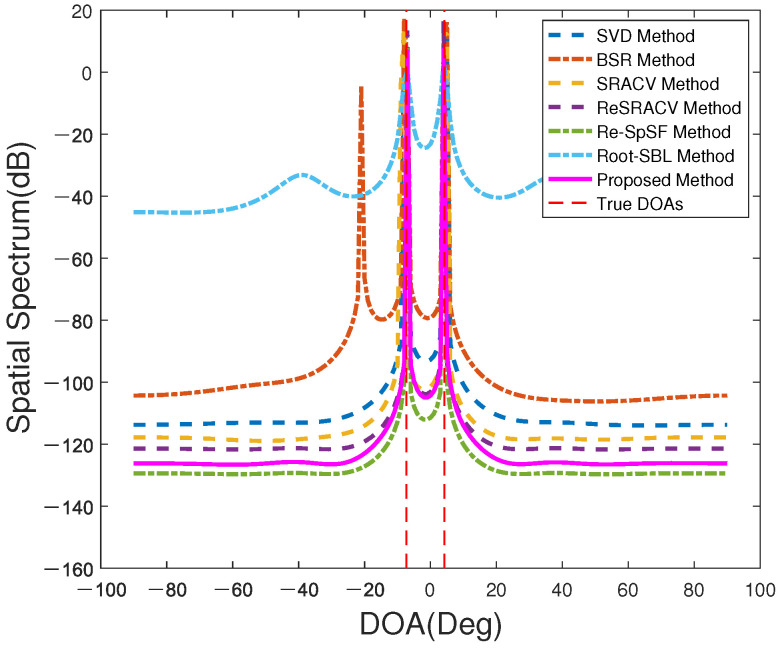
The spatial spectrum of all methods.

Figure 1 describes the spatial spectrum of all estimators with SNR=0 dB and T=200. Additionally, their corresponding estimation results are recorded in Table 2. On the one hand, there are two sharp peaks for these approaches in Figure 1, implying that they are able to maintain direction finding under unknown mutual coupling and off-grid conditions. What is more, it is not hard to find that the peaks of the Root-SBL approach are the least sharp and the sidelobe is the highest, while the peaks of the potential Re-SpSF and the proposed methods are the sharpest and the sidelobes are the lowest. On the other hand, Table 2 depicts the proposed method outperforms the Re-SpSF and ReSRACV algorithms, and is the closest to the real DOAs among these estimators. Since the estimated DOAs of Re-SpSF are the same as those of the ReSRACV, Re-SpSF and ReSRACV frameworks show a similar estimation performance. Conversely, the SVD and BSR algorithms are furthest from the desired DOAs and have the worst performance. Moreover, the Root-SBL estimator is overall closer to the true DOAs than the SRACV framework, but not as close as the Re-SpSF and ReSRACV approaches. Hence, the proposed method is superior to other algorithms in terms of resolution and accuracy.

**Figure 2:**
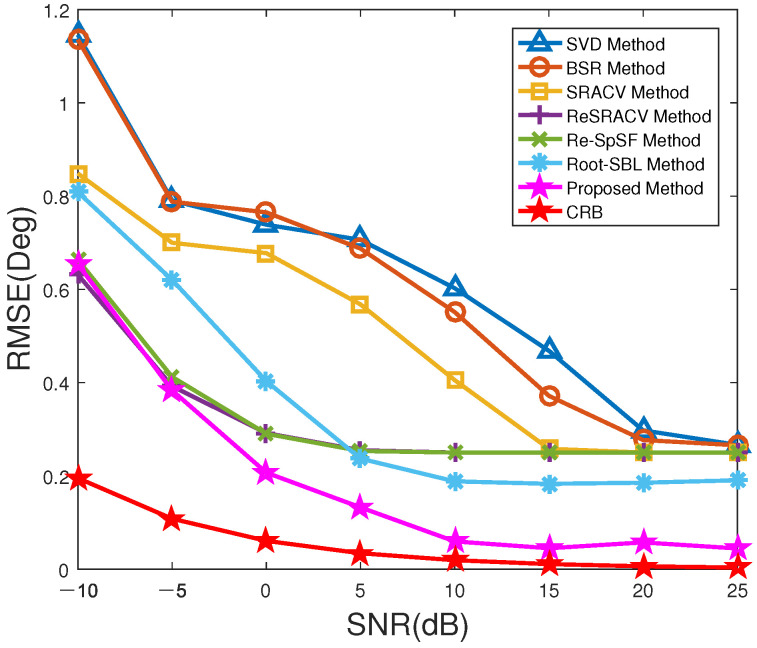
RMSE versus SNR of all methods.

Figure 2 reveals the RMSE versus SNR of all methods with T=200. From Figure 2, with the increase of SNR, the RMSEs of all methods decrease to a certain extent, i.e., these estimators improve the estimation accuracy. Among them, the SVD and BSR methods have the largest RMSEs and the worst performance, where the RMSE of the SVD approach is close to that of the BSR algorithm over the whole interested SNR range. Meanwhile, the RMSE of the SRACV approach is lower than that of them, but higher than that of the ReSRACV and Re-SpSF schemes. That is, the ReSRACV and Re-SpSF estimators are better than the other three approaches in accuracy, mainly due to their reweighted measure. Obviously, the RMSEs for the ReSRACV and Re-SpSF schemes are almost equivalent, revealing that their corresponding estimation performances are very similar. What is more, it can be found that at low SNR (SNR=−10 dB), the RMSE of the proposed estimator is slightly greater than that of the ReSRACV algorithm, but less than that of the Root-SBL and Re-SpSF algorithms, especially the Root-SBL algorithm. As SNR improves, the RMSE of the proposed method is the lowest and closest to CRB. Its estimation performance is far better than that of algorithms such as the ReSRACV, Re-SpSF, and Root-SBL schemes. It should be noted that the difference between Re-SpSF and the proposed algorithm is whether an off-grid representation is performed. The Re-SpSF algorithm does not perform off-grid representation, causing a higher RMSE than that of the proposed method. In other words, thanks to the off-grid representation, the proposed method displays good robustness and satisfactory estimation accuracy for the off-grid error. In general, the proposed method has advantages over the other six approaches.

**Figure 3:**
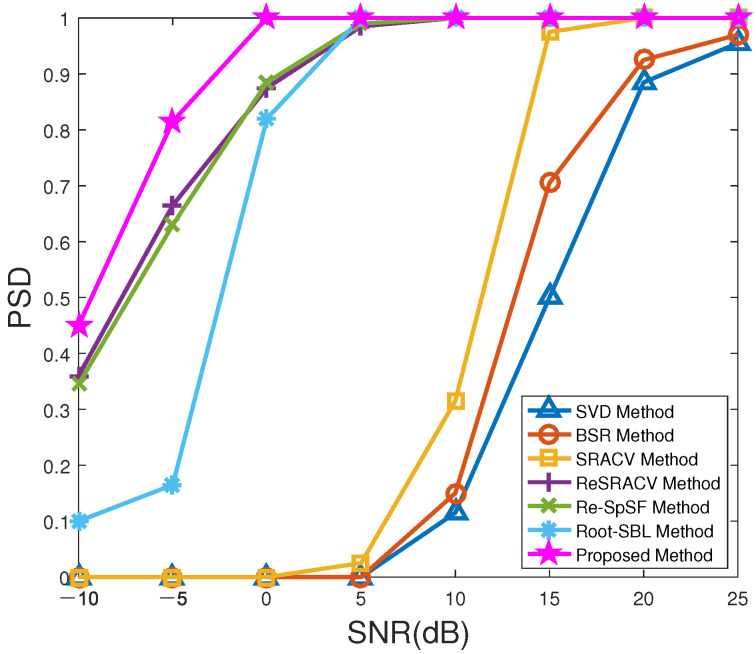
PSD versus SNR of all methods.

Figure 3 demonstrates the probability of successful detection (PSD) versus SNR of all methods with T=200. If the error between the interested DOAs $\theta_{k}$ and the estimated DOAs ${\hat{\theta }}_{k}$ is less than $0.7^{\circ }$, i.e., $|{\hat{\theta }}_{k}-\theta_{k}|<0.7^{\circ }$, the signal source can be considered to be successfully detected. As expected, the PSDs of all estimators gradually increase as SNR improves. More importantly, the PSD of the proposed method not only outperforms that of the other six algorithms over the entire selected SNR range, especially for relatively low SNRs, but also reaches 100% faster than that of the other algorithms.

**Figure 4:**
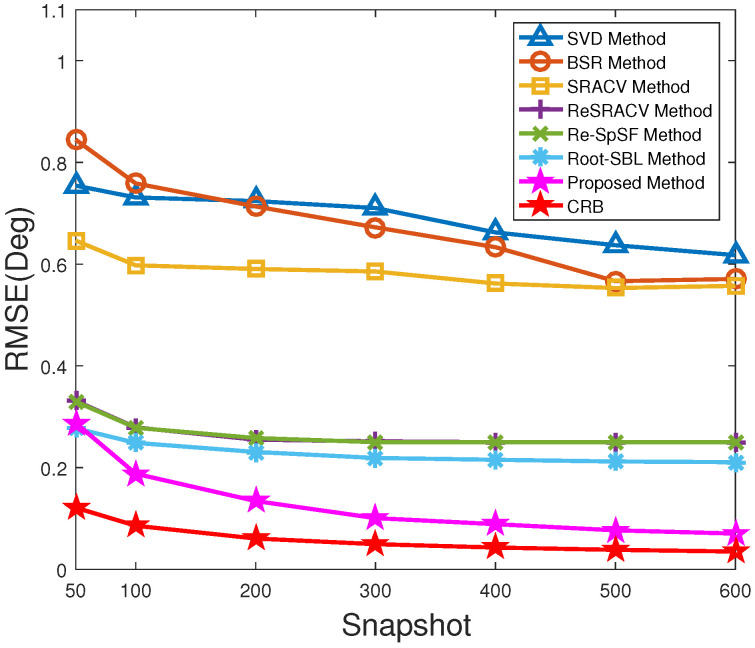
RMSE versus snapshots of all methods.

Figure 4 illustrates the RMSE versus snapshots of all algorithms with SNR=5 dB. Additionally, their corresponding RMSEs are recorded in Table 3. Similar to Figure 2, the increase in snapshots number promotes the estimation performance of these approaches to some extent. Simultaneously, the performance of the SVD approach remains similar to that of the BSR framework, being the worst among these estimators. Furthermore, it is obvious that the RMSE of the ReSRACV framework is almost the same as that of the Re-SpSF scheme and much lower than that of the SRACV estimator. This implies that the ReSRACV and Re-SpSF algorithms have the same estimation performance and outperform the SRACV framework. This is mainly attributed to the fact that the ReSRACV framework uses the same reweightwd measure as the Re-SpSF scheme, which reduces the RMSE well. However, it is not difficult to find from Table 3 that the RMSEs of the ReSRACV and Re-SpSF methodologies stop decreasing after reaching a certain level (i.e., RMSE = 0.2500), indicating that their accuracy no longer improves and tends to saturate in such case. This may be because their discrete grid interval (i.e., $\epsilon =1^{\circ }$) is set too large, resulting in the increase in snapshots not being enough to resist the influence of off-grid error on the estimation accuracy. The RMSE of the Root-SBL estimator is lower than those of the ReSRACV and Re-SpSF schemes, but higher than that of the proposed approach, except for the case of T=50. It means that the Root-SBL methodology is superior to the ReSRACV and Re-SpSF estimators and inferior to the proposed method. Moreover, the RMSE of the proposed algorithm is lower than that of the potential Re-SpSF framework. That is to say, the off-grid representation in the proposed method enhances the robustness to off-grid error well and improves the estimation accuracy. In short, the proposed method takes the lowest RMSE and the best estimation performance, which is closest to CRB. Therefore, the proposed method outperforms the other six estimators.

**Figure 5:**
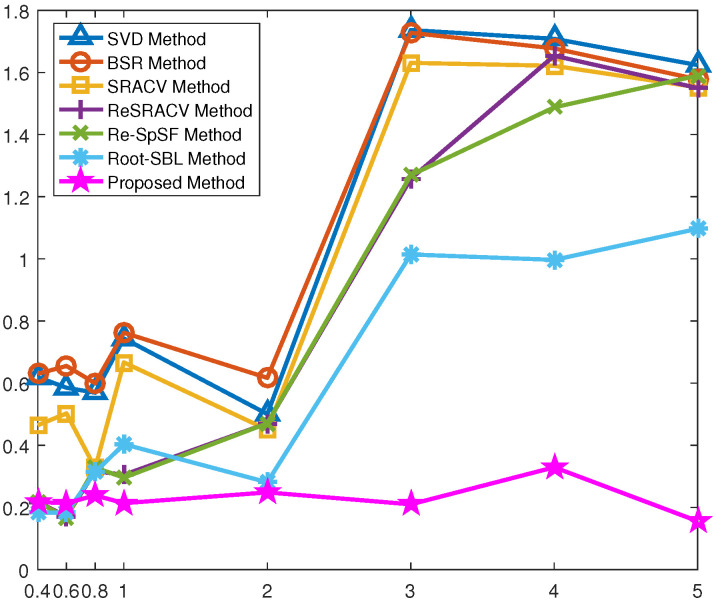
RMSE versus grid interval of all methods.

Figure 5 displays the RMSE versus grid interval of all methods, where SNR=0 dB and T=200. In most of the selected grid intervals, the RMSE of the proposed method is the smallest, closest to 0, and least influenced by the coarse grid intervals. Hence, it shares the best and most stable estimation performance. The main reason is that it not only concerns mutual coupling and off-grid errors to reinforce the robustness, but also exploits the reweighted strategy to improve accuracy. Similarly, the Root-SBL algorithm also considers all factors, unlike the other five regularization methods that focus only on mutual coupling. Clearly, when the grid interval is less than $1^{\circ }$, the RMSE of the Root-SBL estimator is approximately equal to those of the ReSRACV and Re-SpSF approaches, which means that its performance is similar to that of the ReSRACV and Re-SpSF schemes. This is mainly because the influence of off-grid gap is relatively weak in this way, while the reweighted measure in the ReSRACV and Re-SpSF estimators ensures high-precision estimation. As the grid interval increases, the off-grid error dominates among these two errors, and thus the Root-SBL algorithm is significantly better than the ReSRACV and Re-SpSF frameworks at this time. Furthermore, the RMSEs of the SVD, BSR, and SRACV schemes are close, farthest from 0, and most affected by the coarse grid intervals, i.e., their performances are similar, the worst, and the least stable. It is emphasized that the Re-SpSF scheme does not perform off-grid representation compared to the proposed method, so its estimation accuracy is easily affected by off-grid error, especially for coarse grid intervals. Therefore, the proposed method has not only superior estimation accuracy, but also satisfactory robustness to off-grid error.

**Figure 6:**
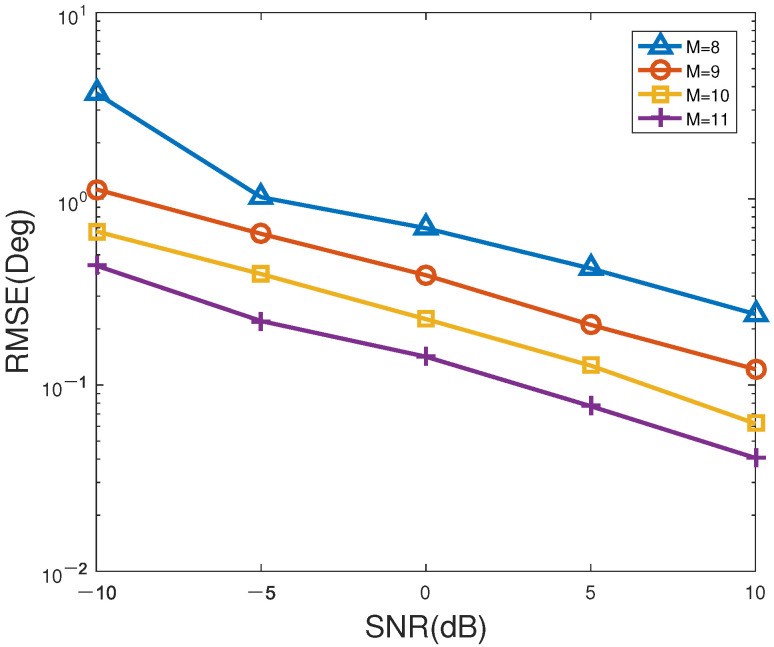
RMSE versus SNR of the proposed method for different number of antennas.

**Figure 7:**
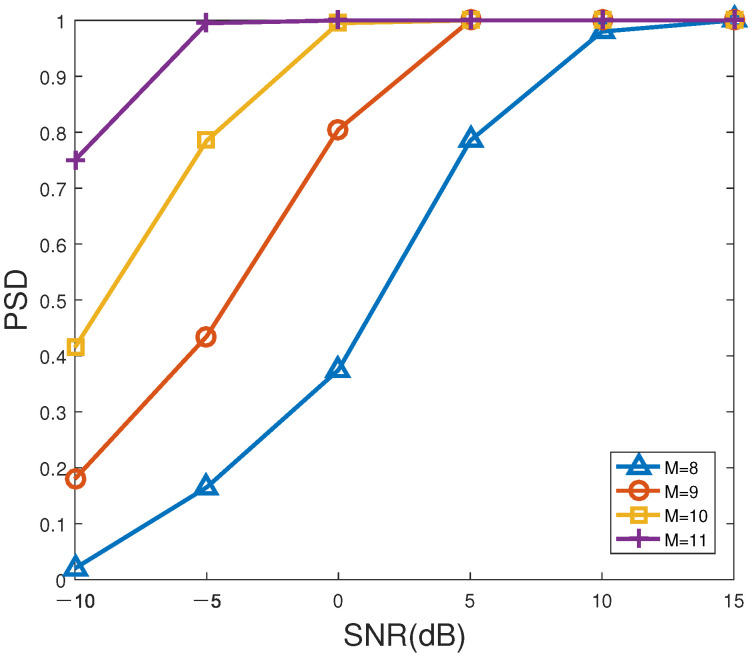
PSD versus SNR of the proposed method for different number of antennas.

Figure 6 and Figure 7 indicate the RMSE and PSD versus SNR of the proposed method for different number of antennas, respectively. In Figure 6 and Figure 7, the number of snapshots is set to T=200. According to Figure 6 and Figure 7, as SNR or/and the number of sensors gradually increases, the RMSE of the proposed method decreases overall, while the corresponding PSD gradually enhances to 100%, especially for unsatisfactory SNR. Evidently, the PSD with M=11 sensors is higher than that of other sensor numbers, which can be the first to reach 100%. M=9 antennas has the lowest PSD, which is the slowest to achieve 100%. Therefore, the performance of the proposed method can be well enhanced by improving the SNR and/or the number of antennas. However, it should be pointed out that using a greater number of antennas to improve accuracy will not only increase the estimation cost, but also aggravate the computational burden, which is not conducive to real-time direction finding. In summary, it is a wise choice for practical applications to choose the appropriate number of antennas for balancing the estimation accuracy and computational load.

**Figure 8:**
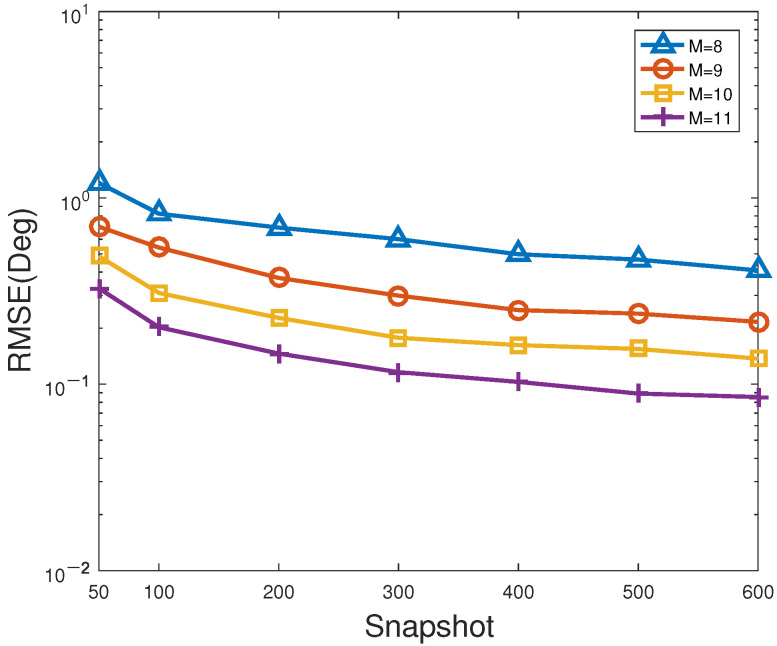
RMSE versus snapshots of the proposed method for different number of antennas.

**Figure 9:**
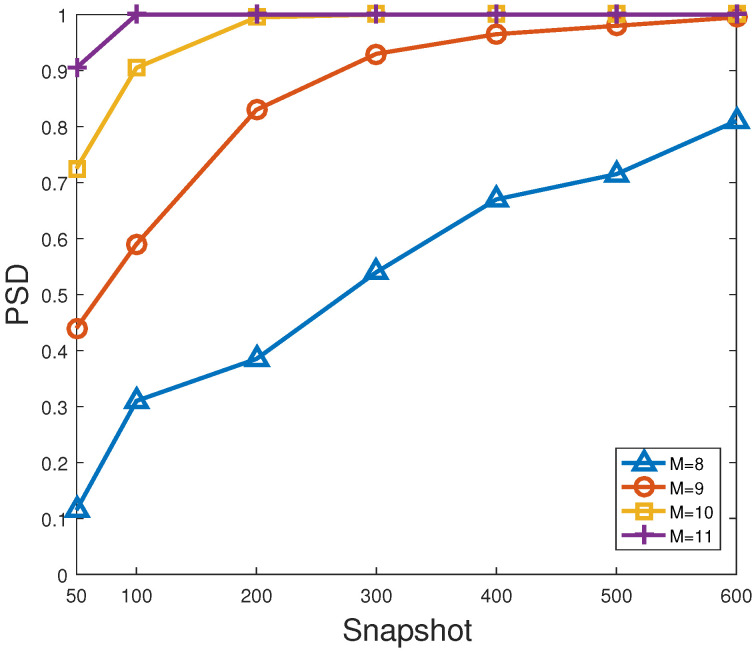
PSD versus snapshots of the proposed method for different number of antennas.

Figure 8 and Figure 9 describe the RMSE and PSD versus snapshots of the proposed method for different number of antennas, respectively. In Figure 8 and Figure 9, SNR is fixed at 0 dB. As shown in Figure 8 and Figure 9, the larger the number of snapshots or/and sensors, the smaller the RMSE of the proposed method, and the larger the corresponding PSD, which behaves like the general trend in Figure 6 and Figure 7. On the one hand, it is feasible for direction finding to ensure estimation accuracy by increasing the number of antennas or/and snapshots. On the other hand, it has to be acknowledged that the larger the number of sensors, the higher the estimation cost, and the heavier the computational complexity. Hence, such superior estimation accuracy attributed to multiple antennas is at the expense of computational load. That is to say, a suitable number of sensors is more applicable to the actual direction finding.

## 6. Conclusions

In this article, an effective and efficient reweighted sparsity-inducing approach achieved by the OGSpSF framework is presented for off-grid DOA estimation in sensor array with unknown mutual coupling. In the proposed method, the decoupled received data is designed by exploiting a selection matrix to escape the unknown mutual coupling disturbance. Then, an enhanced OGSpSF recovery model is constructed by incorporating the linearly approximated off-grid error term into the potential on-grid sparse model to ensure the robustness. Subsequently, an upgraded Re-OGSpSF framework is explored for off-grid DOA estimation using joint sparse recovery, where a MUSIC-like weighted matrix is further implanted to improve accuracy. Eventually, off-grid DOA estimation can be estimated via the spatial spectrum of the reconstructed sparse vector. Attributed to the off-grid representation and reweighted attempt, the proposed method can efficiently provide high-precision continuous DOA estimation by setting a coarse grid interval, making it more suitable for real-time direction finding. Extensive simulation results show the effectiveness and efficiency of the proposed approach.

## Figures and Tables

**Table 1:** Relevant key notations.

Notations	Definitions
${\left(\cdot \right)}^{T}$, ${\left(\cdot \right)}^{*}$, and ${\left(\cdot \right)}^{H}$	Transpose, conjugate, and conjugate-transpose
$0_{M\times K}$	M×K dimensional zero matrix
$I_{M}$	M×M dimensional identity matrix
diag{·}	Diagonalization operator
E{·}	Mathematical expectation operator
⊗ and ⊙	Kronecker and Khatri–Rao products
|·| and det{·}	Absolute value and determinant operators
min{·} and max{·}	Return the minimum and maximum value operators
${∥\cdot ∥}_{0}$, ${∥\cdot ∥}_{1}$, and ${∥\cdot ∥}_{2}$	$l_{0}$-norm, $l_{1}$-norm, and $l_{2}$-norm

**Table 2:** Comparison of estimation results of all methods.

Methods	DOA 1	DOA 2
SVD Method	$-8.0000^{\circ }$	$5.0000^{\circ }$
BSR Method	$-8.0000^{\circ }$	$5.0000^{\circ }$
SRACV Method	$-8.0000^{\circ }$	$4.0000^{\circ }$
ReSRACV Method	$-7.0000^{\circ }$	$4.0000^{\circ }$
Re-SpSF Method	$-7.0000^{\circ }$	$4.0000^{\circ }$
Root-SBL Method	$-7.1965^{\circ }$	$3.7667^{\circ }$
Proposed Method	$-7.3479^{\circ }$	$4.5000^{\circ }$

**Table 3:** RMSE under different number of snapshots of all methods.

		Snapshot	50	100	200	300	400	500	600
	RMSE								
Method									
SVD Method			0.7544	0.7311	0.7240	0.7103	0.6624	0.6372	0.6177
BSR Method			0.8446	0.7587	0.7134	0.6723	0.6333	0.5665	0.5709
SRACV Method			0.6462	0.5978	0.5905	0.5855	0.5620	0.5530	0.5574
ReSRACV Method			0.3313	0.2790	0.2549	0.2517	0.2500	0.2500	0.2500
Re-SpSF Method			0.3298	0.2783	0.2581	0.2500	0.2500	0.2500	0.2500
Root-SBL Method			0.2773	0.2487	0.2307	0.2189	0.2154	0.2120	0.2107
Proposed Method			0.2878	0.1880	0.1343	0.1007	0.0888	0.0767	0.0703

## Data Availability

Not applicable.
